# A Case of Rituximab Use as an Induction and Maintenance of Remission in ANCA-Associated Vasculitis

**DOI:** 10.1155/2016/7275860

**Published:** 2016-02-23

**Authors:** Neveen Awad, Shahd Hafiz, Abdurahman Albeity, Hani Almoallim

**Affiliations:** ^1^Department of Internal Medicine, Medical College, Umm-Al Qura University, Makkah, Saudi Arabia; ^2^Department of Internal Medicine, King Faisal Specialist Hospital and Research Center, Jeddah, Saudi Arabia; ^3^Alzaidi Chair of Research in Rheumatic Disease, Umm-Al Qura University, Makkah, Saudi Arabia

## Abstract

Antineutrophil cytoplasmic antibody- (ANCA-) associated vasculitis (AAV) is a multisystem autoimmune disease affecting mainly microscopic blood vessels due to circulating autoantibodies against neutrophil cytoplasmic antigens. We report a case of a 57-year-old female patient presenting with hemoptysis, sinusitis, and conjunctivitis. Based on lung biopsy, the diagnosis of antineutrophil cytoplasmic antibody- (ANCA-) associated vasculitis (AAV) was established. She was put on rituximab as induction and maintenance therapy. She responded initially to rituximab as induction therapy but failed to respond in the maintenance course of the drug. Rituximab was stopped and mycophenolate mofetil was administered. She responded as laboratory c-ANCA titers turned negative and symptoms subsided. There are no randomized clinical trials addressing rituximab effect in induction and remission at the same time. This case report doubts the efficacy of the use of rituximab therapy for both induction and maintenance of remission at the same time, waiting for the results of the ongoing trials.

## 1. Introduction

Antineutrophil cytoplasmic antibody- (ANCA-) associated vasculitis (AAV) is a multisystem autoimmune syndrome affecting mainly microscopic blood vessels due to circulating autoantibodies against neutrophil cytoplasmic antigens [1]. Cyclophosphamide and glucocorticoids have been the standard of remission induction therapy for severe AAV for 40 years [2]. These are associated with high morbidity rates and adverse effects such as infections, cancer, and infertility [1]. Rituximab is a chimeric monoclonal anti-CD20 antibody [1]. Its use allows reducing the exposure to cyclophosphamide and maintenance immunosuppression [1]. It has been used in AAV for induction of remission as in RAVE and RITUXVAS trials, and in the maintenance as in MAINRITSAN trial [1, 3, 4]. There are no clinical trials addressing rituximab effect in induction and maintenance of remission at the same time. We report a case of ANCA-associated vasculitis where rituximab was used for both induction and maintenance of remission.

## 2. Case Report

A 57-year-old female, not smoker, was admitted on March 29, 2014, with hemoptysis and history of recurrent sinusitis for one year. She had also positive history of recurrent eye inflammation epistaxis, dizziness, and left ear dry perforation. The right ear and audio assessment were normal. There was no arthritis. Other systemic reviews and physical examinations were unremarkable. Chest X-ray showed bilateral chest infiltrates. CT-scan revealed mucosal thickening of the floor of the left maxillary antrum, otherwise normal sinuses, and it also showed collapsed consolidation of the right lung with air bronchogram. The left lung showed ill-defined patches of ground glass and tree in bud appearance. Bronchoscopy showed mild pulmonary hemorrhage. Lung biopsy showed no granuloma but small vessels inflammation. Bronchoalveolar lavage (BAL) was negative for malignancy or any specific lesion.

Laboratory findings revealed high titers of c-ANCA 1 : 40 where it should be absent or of level less than 1 : 20, and p-ANCA was negative. Rheumatoid Factor (RF), ESR, and CRP were elevated of 225 IU/mL, 115 mmHr, and 92 mg/dL, respectively (Table 1). On the other hand, antinuclear antibody (ANA), Hepatitis B antigens, Hepatitis C antigens, and TB AFB and PCR were all negative and complements C3 and C4 were normal. Urine analysis identified hematuria and proteinuria. Serum albumin was low (2.5 g/dL). Renal function tests showed high level of creatinine (299 mg/dL).

She was diagnosed as ANCA-associated vasculitis, most likely granulomatosis polyangiitis, and her Birmingham Vasculitis Activity Score (BVAS) was 10 according to BVAS Version 3 form [5].

Patient was started on Pulse Steroid 1 gm IV for 3 days and then 60 mg orally daily.

On April 28, 2014, she was on Essential D-3 50,000 international units once weekly, Feroglobin capsules twice a day, and folic acid tablets 5 mg once weekly.

She was started on rituximab 1000 mg as induction of remission in May 2014. It was given after 30 minutes of its premedications methylprednisolone 100 mg, diphenhydramine 50 mg per month, and acetaminophen 975 mg per month. It was given each week for two weeks similar to Rheumatoid Arthritis (RA) induction regimen according to REFLEX trial [6].

Methotrexate was given as 12.5 mg orally once per week, but it was shortly tapered afterwards. Prednisone was tapered to 15 mg per month twice a day.

On March 4, 2015, the patient was given the maintenance dose of rituximab according to MAINRITSAN trial. Rituximab was administered at dose of 500 mg IV once weekly for 2 weeks with the premedications mentioned earlier. While infusing, she developed reaction to rituximab appearing as skin rashes and redness over cheeks only. The infusion stopped for 30 minutes and resumed once her vital signs were stabilized with slow rate infusion.

On April 8, 2015, she came complaining of pain and redness in the left eye, and similar symptoms of her initial presentation of the disease. She was diagnosed with anterior scleritis by ophthalmology service. Therefore, she was started on 30 mg of prednisolone. Symptoms were improved and the drug was tapered by 5 mg per week. She was also on calcium and vitamin D, but the plan for continuing rituximab as maintenance therapy was a question mark.

On May 27, 2015, she came complaining of ear pain, decreased hearing, tinnitus in the left side, and bilateral eye redness. That was attributed probably to the tapering of the prednisone as she at that time was on 10 mg once daily. Laboratory c-ANCA titers were positive (18.2). It was considered as rituximab maintenance failure. The plan was to increase the dose of prednisone to 30 mg/day and to add mycophenolate mofetil (MMF) 500 mg twice per day as an alternative maintenance therapy. The dose was increased to 1 gm twice per day to control symptoms. She was hypertensive as well; amlodipine 5 mg once daily was given.

On June 3, 2015, symptoms improved and ANCA titers decreased but hearing was still reduced. MMF dose was increased to 1 gm in the morning and 500 mg in the evening, and prednisone decreased to 25 mg/day. She was still hypertensive; amlodipine was increased to 10 mg once daily.

On July 1, 2015, there was no new complaint and symptoms improved, therefore tapering prednisone 2.5 mg per week according to symptoms.

On August 26, 2015, c-ANCA turned negative and the patient is doing well on the same regimen.

Upon her last followup on October 28, 2015, her prednisone was kept at 10 mg/day and mycophenolate at 1 gm twice a day with no complaint.

## 3. Discussion

Although there are many studies that suggest the use of rituximab as an induction or maintenance therapy, there is a shortage of data about the use of the drug for both targets at the same time for the selected patients and many studies recommend further research.

Two prospective randomized trials (RAVE and RITUXVAS) of induction of remission confirmed that rituximab can be effective as an induction therapy for active AAV [5]. In RITUXVAS, rituximab based regimen was not superior to the use of standard cyclophosphamide, and the adverse effects and mortality rate did not differ between the two groups significantly [1]. In RAVE, approximately the same results were obtained regarding the mean duration of complete remission and the adverse effects, with fewer episodes of severe leukopenia in the rituximab group [3]. Also the relapses were equal in the two groups which were rare in the absence of both B cells and ANCA. However, rituximab based regimen was superior in patients who had relapsing disease at 6 months [3].

Regarding the use of rituximab in remission maintenance, MAINRITSAN trial compared rituximab with azathioprine and it showed that rituximab is superior to azathioprine for maintaining remission [4]. Another retrospective study of rituximab for remission maintenance seemed to be successful in complete or partial remission of AAV [7]. On the contrary, there is a study addressing rituximab use for the long term in ten AAV patients, whose disease was either refractory or relapsing [8]. It resulted in sustained responses but unfortunately 25% suffered severe adverse effects and 50% were relapsing [8]. A retrospective study used a single-dose rituximab for remission induction and maintenance therapy in AAV and concluded that the combination of single-dose rituximab with other immunosuppressant agents seems to be less effective than the standard rituximab regimen for the induction of remission of AAV [9]. Again there is a lack of conclusion as the duration of followup is not sufficient for the course of the disease which is relapsing and remitting in its nature.

Considering all these studies, concerns were raised on how effective rituximab can be when it is used as induction and maintenance therapy at the same time. Our patient improved initially and became ANCA negative after 2 months of the drug induction therapy and remained in remission for 7 months. On the other hand, after the administration of the maintenance dose she had relapses, as she developed the same presenting symptoms again, ear symptoms and hypertension.

The weak response to the maintenance therapy with rituximab can be due to a variety of factors. The multiorgan affection and the patient age may have played a role. In addition, probably the dependent nature of this disease on prolonged doses of steroids may have also contributed to relapse. Mycophenolate showed good efficacy in maintaining remission and as steroid sparing agent in our case as shown in other studies [10].

We doubt the efficacy of using rituximab as induction and maintenance therapy at the same time in AAV according to our case report. This patient clearly failed to respond well on the long term. Rituximab may be as effective as the traditional treatment for induction therapy or for maintenance. The concomitant use of it in both induction and maintenance therapy at the same time needs further evaluation.

## Figures and Tables

**Table 1:** 

Test	Patient's result	Normal range
c-ANCA	1 : 40	<1 : 20
p-ANCA	<1 : 20	<1 : 20
RF	225 IU/mL	<15 IU/mL
ESR	115 mm/hr	0–30 mm/hr
CRP	92 mg/dL	0–10 mg/dL
Creatinine	299 micromol/L	60–120 micromol/L
